# Developing a Framework for Online Review-Based Health Care Service Quality Assessment: Text-Mining Study

**DOI:** 10.2196/66141

**Published:** 2025-07-09

**Authors:** Xue Zhang, Jianshan Sun, Xin Li, Yezheng Liu, Chenwei Li

**Affiliations:** 1 Fuyang Normal University, Fuyang, China; 2 Hefei University of Technology, 193 Tunxi Road, Hefei, 230009, China, 86 18956561661; 3 Key Laboratory of Philosophy and Social Sciences for Cyberspace Behaviour and Management, Hefei, China; 4 City University of Hong Kong, Hong Kong, China (Hong Kong); 5 Xi’an Jiaotong-Liverpool University, Suzhou, China

**Keywords:** health care service, service quality, online reviews, text mining, SERVQUAL

## Abstract

**Background:**

With the development of online health care platforms, patient reviews have become an important source for assessing medical service quality. However, the critical aspects of quality dimensions in textual reviews remain largely unexplored.

**Objective:**

This study aims to establish a comprehensive medical service quality assessment framework by leveraging online review data. Such a framework would support large service providers, such as online platforms, to assess the quality of many doctors efficiently.

**Methods:**

We adopted a text-mining approach with theory-driven topic extraction from online reviews to develop a service quality assessment framework. The framework is based on topic and sentiment classification methods. We conducted an empirical analysis to assess the validity of the framework. Specifically, we examined if patients’ sentiments regarding our extracted dimensions affect demand (number of consultation requests) due to quality signals reflected in these dimensions.

**Results:**

We develop a 5-dimensional health care service quality framework (HSQ-5D model). In the empirical study, patient demand is affected by these dimensions, including expertise (coefficient=1.12; *P*<.001), service delivery process (coefficient=5.60; *P*<.001), attitude (coefficient=0.82; *P*<.001), empathy (coefficient=2.65; *P*<.001), and outcome (coefficient=0.26; *P*<.001; through patients’ perceived quality from reviews). The 5 dimensions can explain 85.52% of the variance in patient demand, while all information from online reviews can explain 85.67%. The results show the validity and the potential practical value of the proposed HSQ-5D model.

**Conclusions:**

This study explores how online reviews can be used to evaluate health care services, offering significant implications for health care management. Theoretically, we extend existing service quality frameworks by integrating text-mining analysis of online reviews, thereby enhancing the understanding of service quality assessment in the digital health context. Practically, the framework can allow health care platforms to identify and reveal doctors’ service quality to reduce patients’ information asymmetry and strengthen patient-provider relationships, ultimately contributing to a more effective and patient-centered health care system.

## Introduction

### Background

The advent of the internet has precipitated a revolutionary transformation of medical service acquisition [1]. Online health care platforms have emerged as pivotal sources that provide the public with unparalleled access to medical resources at a reduced cost [2]. These platforms facilitate closer connections between patients and health care professionals [3] and empower patients through textual messages, phone calls, and video calls [4]. These services serve as viable alternatives to traditional face-to-face consultations and provide timely services when needed [5]. Although existing research has yielded important insights into how online consultations enhance the relationship between patients and doctors [6], reduce medical expenses [7], and enhance public well-being [8], a notable gap persists in measuring the service quality of online health care services. Small-scale service quality assessment, including in-person medical practices, online health care platforms, and telemedicine, can be conducted through questionnaires and interviews [9-11]. However, these approaches are costly and time-consuming to scale up [12] and, hence, do not suit the needs of online health care platforms with millions of doctors and patients.

### Health Care Services Assessment

Evaluating health care services has always been a complex task [13], with existing literature offering various perspectives to define and assess the quality of health care services. Early studies focused on fundamental aspects such as accessibility, effectiveness, and continuity of health care services, using these as standards to assess service quality [14]. Similarly, Carman [15] identified 6 indicators of service quality: tangibles, reliability, safety, empathy, convenience, and cost of health care services. Bowers et al [16], however, proposed a slightly different set of 6 factors, emphasizing reliability, responsiveness, communication, accessibility, understanding, and consideration. In addition to these individual factors, some scholars have argued that healthcare service quality should be evaluated from a broader multiple-stakeholder perspective, incorporating patient-centered factors and disease characteristics, treatment types, functionality, and the roles of health workers [17].

A widely used framework to depict patients’ assessment of health care services is Donabedian’s structure-process-outcome (SPO) framework [18,19]. In this model, the structure refers to the attributes of health care providers, such as hospital rankings [20,21], doctor qualifications [22], whether doctors are part of an expert team [23], and doctors’ demographic characteristics [24]. The process concerns patients’ experiences during health care services, including factors such as the communication atmosphere [25] and examination and treatment plans [26]. The outcomes focus on the consequences of delivering health care services, such as fatality, disabling, and recovery rates of the hospital [27].

Aligned with the SPO framework, the SERVQUAL (service quality) model (oriented from the retailing industry) has become one of the most widely recognized tools for measuring user perceptions of service quality [28]. This model identifies 5 key dimensions essential for evaluating and enhancing service quality in various sectors: tangibility (availability and accessibility of physical resources, facilities, and equipment), reliability (ability to deliver services appropriately); responsiveness (willingness to offer prompt services), assurance (credibility and security to inspire confident outcomes), and empathy (understanding and concern for each patient). The fundamental principle underlying the SERVQUAL model is recognizing the disparity between the services rendered by providers and the level of service expected by consumers [29]. Identifying these disparities presents a notable opportunity to enhance the quality of health care services [30].

### Online Platforms’ Need for Service Quality Assessment

Although many service quality assessment frameworks exist, in practice, they are often applied through questionnaires and interviews [9-11]. With the development of online health care platforms, patients must assess the quality of doctors in real time [31]. The literature has explored how information available on online health care platforms serves as quality signals, which can be classified as structured and unstructured signals.

Structured information, including ratings, recommendation scores, and reputation scores given by patients, has long been recognized as an important factor in providing patients with a quick way to assess the perceived quality of a doctor [32,33]. Similarly, the number of patient votes, letters of acknowledgment, and virtual gifts have also gained importance in reflecting the quality of medical services and the trust between patients and doctors [22,34]. In addition, the activity level of doctors, as reflected in previous consultation records, serves as a direct indicator of service quality. For example, doctors offering health care services through multiple channels tend to increase availability [4]. Doctors who voluntarily share articles and provide free health care consultation services also signal the quality of their services to the public [35].

Furthermore, unstructured information, particularly from online reviews, provides more detailed insights into a doctor’s quality assessment, provides direct evidence of service quality, and reflects the nature of doctor-patient interactions [36]. These reviews represent voluntary sharing of health care experiences, perceptions of service quality, and satisfaction metrics from various consumer-perspective dimensions [37]. However, many previous studies have primarily focused on the structured characteristics of online reviews. For instance, some researchers have examined the impact of review length and volume on patient perceptions [38], whereas others have explored the overall sentiment of online reviews, suggesting that positive sentiment is correlated with a higher likelihood of quality signals [39,40].

These quality signals are critical in helping patients make more informed choices, which, in turn, drives the demand for healthcare services on the platform [7]. Doctors perceived to have higher quality based on structured signals such as ratings, and unstructured signals, including detailed reviews, are more likely to be selected by patients [40]. Nevertheless, a critical knowledge gap persists regarding the transformation of these signals, particularly unstructured signals, into a structured framework within the quality assessment literature.

### Textual Analysis–Based Quality Assessment

Text mining is a method of converting unstructured textual information into structural information. Recent advancements in machine learning and artificial intelligence (AI) have significantly improved unstructured data processing. Several studies have confirmed the efficiency of machine learning tools in online review mining for quality measurement in mobile banking [41], the hospitality industry [42-44], the airline industry [45], the tourism sector [46], and the food delivery services [47,48]. In particular, latent Dirichlet allocation (LDA), sentiment analysis, and various deep learning methods have been used to extract and discern customer opinions and sentiment polarity on quality dimensions [41,43-45], and identify essential factors that influence customer satisfaction [46,48].

Inspired by these developments, unstructured patient feedback is used in online health care platforms, presenting an emerging opportunity to apply machine learning and AI techniques to obtain deeper insights into patient experiences [49]. For example, scholars have applied topic modeling and deep learning models for sentiment classification to analyze COVID-19-related discussions on social media [50,51], providing valuable insights into public health concerns. Topic modeling and sentiment analysis have also been used to identify key patient concerns and experiences with doctors and care [52,53]. Machine learning methods such as convolutional neural network and long short-term memory were also used to classify the quality of health care service delivery as “high vs low” [54]. However, these studies have often focused on a specific application and relied on broad sentiment categories, which do not provide enough granularity to understand the specific elements influencing patient satisfaction. As a result, they fail to adequately assess the multidimensional nature of health care service quality. They have not investigated the development of a comprehensive framework for service quality assessment using online reviews. The proposed approach, which accounts for complex, multidimensional feedback and to classify the quality of health care services in a more granular manner represents a significant advancement over previous approaches.

### Research Gap

Service quality assessment is highly critical for health care services; nevertheless, existing quality frameworks, such as Donabedian’s SPO model and the SERVQUAL model, primarily focus on traditional health care settings, including hospital infrastructure, doctor qualifications, and physical resources. These models do not account for the unique aspects of online health care platforms, where digital interactions replace the physical infrastructure. This highlights the need for a dedicated service-quality framework tailored to online health platforms.

As online health care platforms become more prevalent, online reviews provide a comprehensive and evolving source of feedback on services [55], offer deeper insights into users’ perceptions [56], and are publicly accessible [57], making them a perfect source for quality assessment. However, the current service quality frameworks lack the sophistication to leverage these sources fully. A challenge in using online reviews to assess medical service quality is their unstructured nature and large volume of content. While the extraction of structured information from online reviews is feasible, as demonstrated in retail and hospitality applications, there remains a significant gap in understanding the diverse aspects of patient unstructured feedback in improving the quality of online health care services.

### Study Aims

This research aims to establish a comprehensive framework for evaluating medical service quality based on online review data, addressing key gaps in the current literature. For this purpose, we begin by using an unsupervised approach to cluster online reviews into distinct topics, allowing us to capture the breadth of patient feedback. These topics are aggregated into quality dimensions using a theory-driven methodology, resulting in a 5-dimensional framework including doctors’ expertise, communication attitude, service delivery process, outcome of services, and empathy, which we call the HSQ-5D (5-dimensional health care service quality framework) model. Following the establishment of the framework, we conduct detailed aspects classification and sentiment calculation on patients’ reviews to evaluate critical service quality dimensions.

To validate the effectiveness of the proposed framework, we conduct a rigorous econometric evaluation, ensuring that the framework’s results are reliable and actionable. Our evaluation shows that the 5 dimensions of the framework capture extensive information from all the reviews, significantly influencing patient demand. By offering a data-driven approach to medical service quality assessment, the study seeks to provide valuable insights for health care providers and platform designers to enhance patient experiences, improve service delivery, and support better decision-making processes. The techniques we use are generic across languages and cultures, ensuring the framework’s generality.

## Methods

### Framework Development Process

#### Overview

We propose a text-mining approach to develop a health care service quality assessment framework. We assumed a dataset of patient reviews and conducted topic modeling to categorize the reviews into distinct thematic clusters. Next, we inspected the clustering results by considering theories on service quality and refined the topics to a smaller number of meaningful dimensions. This framework can be applied to real-world settings through text classification and sentiment calculation. To ensure the framework’s validity, we conducted an empirical study to examine whether its dimensions affect patient demand, assuming that patients extract quality signals from online reviews to guide doctor selection. By analyzing how the dimensions correlate with demand, we assess whether they reflect patients’ key decision-making factors regarding medical service quality. A significant relationship would demonstrate that the framework captures the critical drivers of patient choice, validating its effectiveness and practicality in quality assessment.

#### Topic Clustering

In this research, we used a widely used LDA method to extract topics from users’ after-service comments [58]. Owing to its stability and intuitive interpretability this analytical approach has gained significant traction in analyzing unstructured textual data such as online reviews and news articles [59]. One of the issues in topic modeling is estimating the optimal number of topics. To address this problem, according to existing solutions, we adopted 3-fold cross-validation to determine the optimal number of topics for our LDA model. For each fold, we trained the model and evaluated its performance by calculating perplexity and likelihood. Perplexity measures how well a model predicts unseen data, with lower values indicating better generalization. Likelihood assesses the model’s fit to the data, with higher values indicating better alignment with the observed data. By comparing these metrics across different topic numbers, we selected the model that achieved the best balance between fitting the training data and generalizing it to the unseen data.

In addition, 2 researchers independently annotated the topics derived from the LDA model to ensure the reliability and accuracy of the topic extraction process. Their annotations focused on identifying the underlying semantic themes and determining whether topics were semantically similar or overlapping. We evaluated the consistency of the coders by calculating the cosine similarity between each coder’s labels for each topic. Given the focus of this study, which aimed to extract more general thematic information rather than medical-specific terms, we further refined the topics by merging similar ones and excluding medical-specific terminology.

#### Topic Refinement

To map topics into service quality themes effectively, we used a theory-driven refinement process based on existing theoretical frameworks. Extending the structure-process-outcome framework and SERVQUAL model, we built an HSQ-5D model based on the summarized topics. Subsequently, we searched the literature to assess the validity of the dimensions of the model.

#### Application

In real-world applications, text and sentiment classification must be conducted according to the dimensions for applying the framework. First, raw online review texts must be segmented into clauses by analyzing semantic context and punctuation. Specifically, we developed an automatic procedure to identify natural clauses within a sentence according to the punctuation marks to determine appropriate boundaries, ensure that each clause maintains its intended meaning in isolation, and split the text into separate units.

Next, we recruited domain experts to code a training set on clauses and aligned them with the dimensions of the framework. All clauses were classified into different quality dimensions using a supervised classification approach called FastText [60]—an efficient deep learning-based supervised text classification model [61]. The trained model can be applied to process all online reviews posted by patients and map them into distinct aspects.

Subsequently, we adopted a lexicon-based sentiment calculation approach to assess whether each clause contained positive or negative patient experiences. We used dictionary-based sentiment computation for the following reasons. First, dictionary-based sentiment computation techniques possess robust interpretability and transparency [62]. Furthermore, using a dictionary method enables us to conform more effectively to the specific context and specialized language of a subject [63]. In health care, the unique medical jargon and patient emotions require tailored approaches. The use of a dictionary-based method improves the adaptability and scalability of the sentiment lexicon, ensuring the accuracy and flexibility of the model. Based on the lexicon, the algorithm marked the position and value of adjectives, adverbs, and negative words in each clause and calculated them into a total point. The final sentiment value was averaged using the number of words in each corpus for normalization.

After deriving the sentiment of each clause, we aggregated the sentiment value for each doctor in each quality dimension, which delivers a service quality score for each dimension.

#### Evaluation

After developing the service quality framework, we evaluated it by examining whether patients’ opinions of the dimensions would affect the selection of doctors. We assumed that patients would extract quality signals from reviews to direct their choices. Therefore, if the extracted dimensions can predict patients’ choices, they should be correlated with human-perceived service quality. Here, we used demand as a proxy for the aspects that represent patients’ choice of doctors. As the 5 dimensions only represent objective aspects of patient reviews, it is necessary to assess whether their opinions are positive or negative (ie, sentiment), which reflects their perception of service quality [64]. When customers exhibit a positive emotional response to a particular service performance, they are more likely to evaluate the service quality favorably [65]. Conversely, negative emotional expressions tend to result in less favorable evaluations [66].

**Figure 1.**
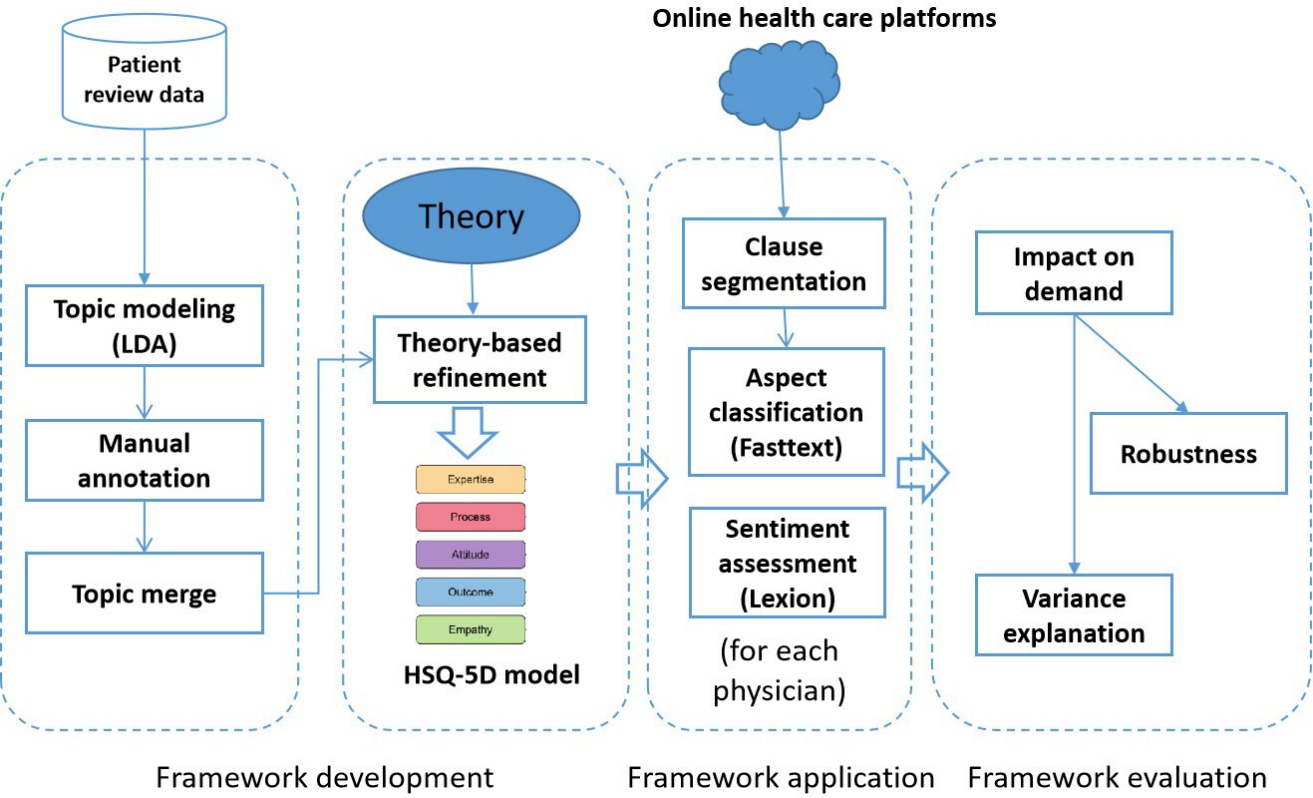
Process for framework development. HSQ-5D: 5-dimensional health care service quality framework; LDA:

Figure 1 summarizes and illustrates the above framework.

### Framework Instantiation

#### Research Context and Data

Data were collected from Haodf.com, one of the largest health care platforms in China, which hosts tens of thousands of registered medical professionals offering online health care services to the public. These services include online registration, image consultation, telephone consultation, video consultation, private doctors, etc. On the platform, patients can choose specific doctors to initiate their consultations and write reviews about their experiences with the services. Compared to health care services in offline settings, patients can easily access doctors regardless of physical distance and gather information about service quality in advance through online reviews, which provides an ideal research context for our study.

Data were crawled weekly from January to March 2023. We selected 10 representative diseases on the platform for analysis, including 5 chronic diseases (diabetes, hypertension, coronary heart disease, stomach cancer, and lung cancer) and 5 nonchronic diseases (lithiasis, menstrual disorders, tonsillitis, colds, and enteritis). The choice of chronic diseases was driven by their long-term nature, ongoing management requirements, and heightened sensitivity of patients to service quality when selecting a doctor. Conversely, the nonchronic diseases were selected for their acute onset and the need for rapid diagnosis and treatment. This disease selection ensured a diverse and representative sample and provided sufficient cases for robust statistical analysis.

Related reviews from patients about these diseases and information about the doctors were collected, which initially gave 6487 doctors and 764,334 reviews. Doctors who had never received any review were excluded, as they do not offer a representative sample of patient feedback, which could distort the findings. In addition, inactive doctors who failed to provide health care services for more than 1 month were excluded as their data may be outdated and incomplete for panel analysis, potentially misrepresenting the current state of health care services. Other relevant information about doctors, such as the type of hospital (general or specialist), the grade of the hospital, the designation of doctors, the quantity of doctors’ homepage visits, patient reservations, doctors’ ratings, and the content of textual reviews, etc, were extracted from both the hospitals’ main pages and the individual doctors’ personal homepages. Finally, 672,195 reviews on 4925 doctors were obtained.

#### Patient Review Topic Extraction

We preprocessed the review texts by cleansing, removing stopwords, and tokenization. We then applied LDA to extract the topics. The LDA iteration results are displayed in Figure S1 in the Multimedia Appendix 1. The line charts indicate that if we set the number of topics at 70, the LDA model exhibits the best performance, achieving minimum perplexity and maximum likelihood, demonstrating an optimal fit between the model and the data with minimal uncertainty. In the subsequent steps of our analysis, we initially executed a 70-topic LDA model with 1000 Gibbs sampling iterations.

A total of 2 researchers independently annotated 70 topics derived from the LDA model. We calculated the cosine similarity based on the text of the labels provided by the 2 annotators. The overall similarity score of 0.8652 indicates a high level of consistency between the coders’ topic labels. It is found that several topics appeared to represent specific types of diseases, such as obstetrics and gynecology issues, intestinal and stomach diseases, and facial organ-related topics. As the study aimed to capture general, overarching themes rather than specific medical terminology, it was necessary to refine and adjust the topics to better align them with the study’s objectives. To merge topics, we employed semantic consistency and word distribution overlap as refinement criteria. First, if the cosine similarity between two topics exceeded a predefined threshold (0.75), indicating that the topics were highly similar in terms of word distribution, we considered merging them. Second, 2 additional researchers were tasked with excluding topics representing diseases, hospital names, and medical terminology.

With these efforts, we nailed down the topics to 12, which were generally more relevant to quality. We provided manual labeling of the topics based on frequently occurring words within each topic [46], which is summarized in Table S1 in the Multimedia Appendix 1. For instance, keywords in Topic 1, such as “expertise,” “skill,” and “specialist,” suggest a focus on technical and professional skills within a medical context, leading to the name “Operational Expertise.” Similarly, the presence of “plan,” “examine,” “treat,” “approach,” and “response” in Topic 5 reflects the systematic methods used in patient care. Thus, it is named as “Care Approach.”

#### Theory-Based Refinement

##### Overview

To map the topics into service quality themes effectively, we employed a theory-driven refinement process based on existing theoretical frameworks. Extending Donabedian’s SPO framework and the SERVQUAL model, we built an HSQ-5D model that encompassed 5 dimensions: doctors’ expertise, service delivery process, outcome of services, communication attitude, and empathy. The HSQ-5D model adopts a patient-centered approach and includes the dimensions of communication attitudes and empathy. The iterative comparison between the 12 topics and the theoretical constructs of the study process involved systematically aligning each topic with the predefined constructs or dimensions of the research. Topics that were semantically similar or aligned with related constructs were merged. Through this careful alignment and merging process, the 12 initial topics were refined into 5 core models that better represented the key dimensions of the study, ensuring theoretical relevance and practical applicability. The final mapping annotations of the 2 researchers between the dimensions and topics are presented in Table S2 in the Multimedia Appendix 1. It is evident that the 2 researchers independently provided identical annotations. Below, we elaborate on the justification for these 5 dimensions.

##### Expertise of Doctors

Doctors’ expertise, defined as their professional competence and medical skills [67], directly influences health care quality and patient choice. Professional competence encompasses a doctor’s qualifications and achievements [68], while medical skills affect health care service quality [69]. Experienced doctors with systematic medical knowledge are more likely to deliver accurate diagnoses and effective treatments [70]. Thus, doctors’ expertise represents an important aspect of service quality in online reviews, which may affect patients’ demands for doctors.

##### Process of Service Delivery

Health care service delivery, which encompasses activities such as examinations, diagnoses, and treatments [71], requires doctors to follow regulated procedures for effective care. The quality of health care depends on an accurate diagnosis, appropriate treatment, and patient care delivery [72]. Inadequate attention can lead to medical carelessness and can influence patient decisions. High-quality medical service delivery results in increased patient satisfaction and recommendations [73]. Thus, doctors’ service delivery processes would represent a crucial part of service quality in online reviews, which may affect patients’ selection of a doctor.

##### Communication Attitude of Doctors

Doctors’ communication attitudes, including their tone and approach to patients, significantly affect patient stress, satisfaction, and treatment perception [74]. Patients are sensitive to doctors’ expressions and prefer to receive care with a positive attitude [75]. A positive communication style can alleviate patient anxiety, enhance doctor-patient interactions, and facilitate health care delivery [76]. Patience and a supportive demeanor in doctors boost patient confidence and treatment optimism [77]. Conversely, impatience or negative attitudes increase patients’ psychological distress [78]. Thus, a doctor’s communication attitude represents an important part of service quality in online reviews, which may affect the number of patients choosing a doctor.

##### Empathy of Doctors

Empathy among doctors can be divided into 2 types: cognitive (understanding conditions) and emotional (responding to emotional demands) [79]. Empathy can be a concern for trustworthiness and significantly affects consumer willingness and satisfaction [80], particularly benefiting those with chronic conditions by reducing depression and anxiety [81,82]. Empathetic doctors are deemed more trustworthy, foster stronger patient relationships, and reduce conflicts [79]. Thus, doctors’ empathy represents an important aspect of service quality in online reviews, which may affect the number of patients who choose the doctors.

##### Outcome of Services

Health care outcomes are measured by hospital metrics such as discharge and readmission rates at the macro level and by patient well-being, including physical, psychological, and physiological status at the individual level [19]. Treatment outcomes significantly influence patient satisfaction [83]. Patients seek health care with specific health objectives in mind [84], and their feedback often reflects their satisfaction or dissatisfaction based on whether these objectives are met. Thus, the service outcome represents an important part of service quality in online reviews, which may affect the number of patients who choose a doctor.

**Figure 2.**
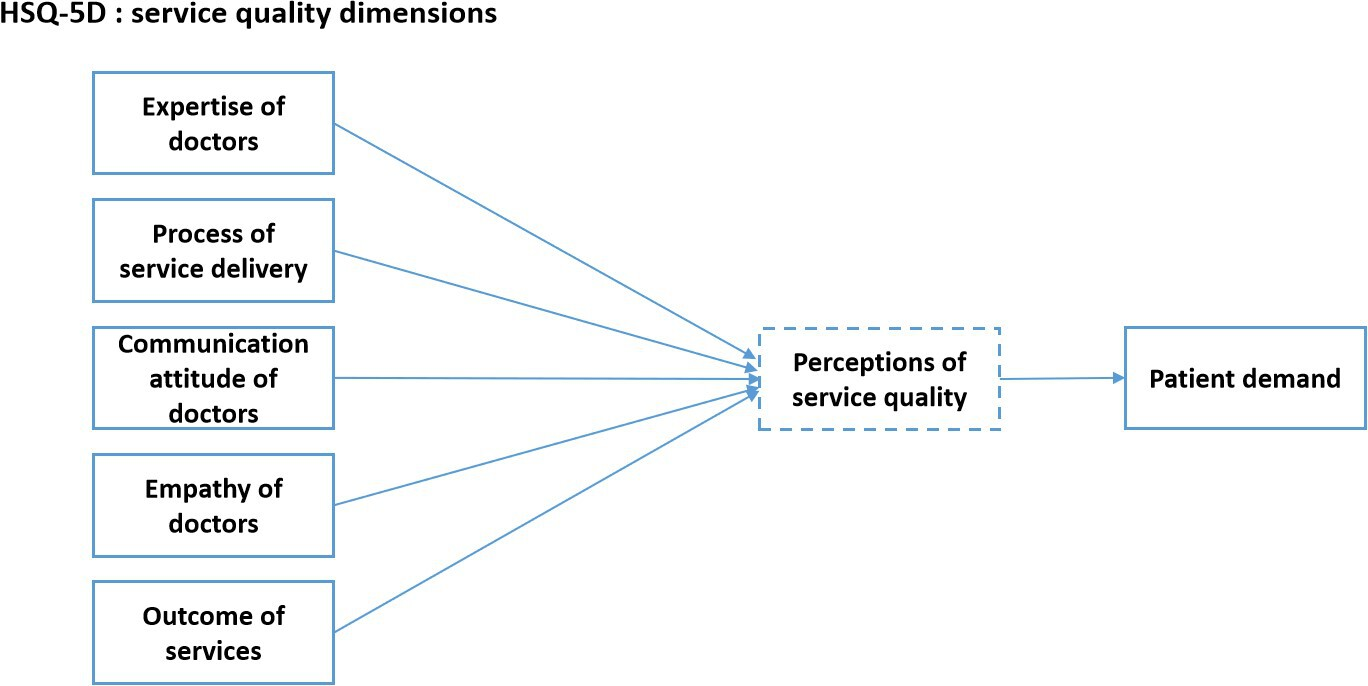
Conceptual framework. HSQ-5D: 5-dimensional health care service quality framework.

Figure 2 shows our research conceptual framework.

### Framework Evaluation

#### Econometric Model

While the HSQ-5D model identifies critical quality dimensions, an empirical analysis helps determine whether these dimensions actually drive patient preferences when selecting doctors. Besides, by evaluating the model in a real-world setting, we can assess whether the quality dimensions hold predictive power beyond theoretical constructs. Consequently, we built doctor-week level panel data regarding the quality dimensions and demands of a focal doctor, followed by further regression analysis. Specifically, for each doctor *i* in each week *t*, we have:


(1) $$Y_{i,t}=\alpha +\Gamma X_{i,t-1}+{\text{Ctrl}}_{i,t}+\theta_{t}+\mu_{i}+\varepsilon_{i,t}$$

where *Y_i,t_* represents the demand for doctor *i* in week *t. X_i,t-1_* are the sentiments of the dimensions of service quality in week *t-1*, representing the patients’ perception of the doctor’s expertise, communication attitude, service delivery process, service outcome, and empathy, respectively. *Ctrl_i_* represents the control variables. *θ_t_* is the time-fixed effect capturing the market trend. *μ_i_* is the individual fixed effect that captures user heterogeneity. *Ε_i,t_* is the random noise.

A few variables were included as controls for confounding factors. First, the professional ranking of hospitals and doctors was controlled, including the type of hospital, city of hospital, grade of hospital, and designation of the doctor. Second, doctors’ activities were controlled, including the number of articles posted by a doctor, which may interfere with patient judgment. Third, other variables, such as the doctor’s ratings, the number of votes received by the doctor, the number of gifts received by the doctor, the number of thanks-you notes received by the doctor, and the number of visits to the doctor’s personal homepage, were also controlled. We add these control variables as these factors may be related to perceptions of service quality or impact patient judgment. In addition, to address the issue of skewed distribution of control variables, we used a natural logarithm for the count variables in the model. We also clustered the errors at the doctor level for a more robust estimation.

#### Aspect Classification

**Figure 3.**
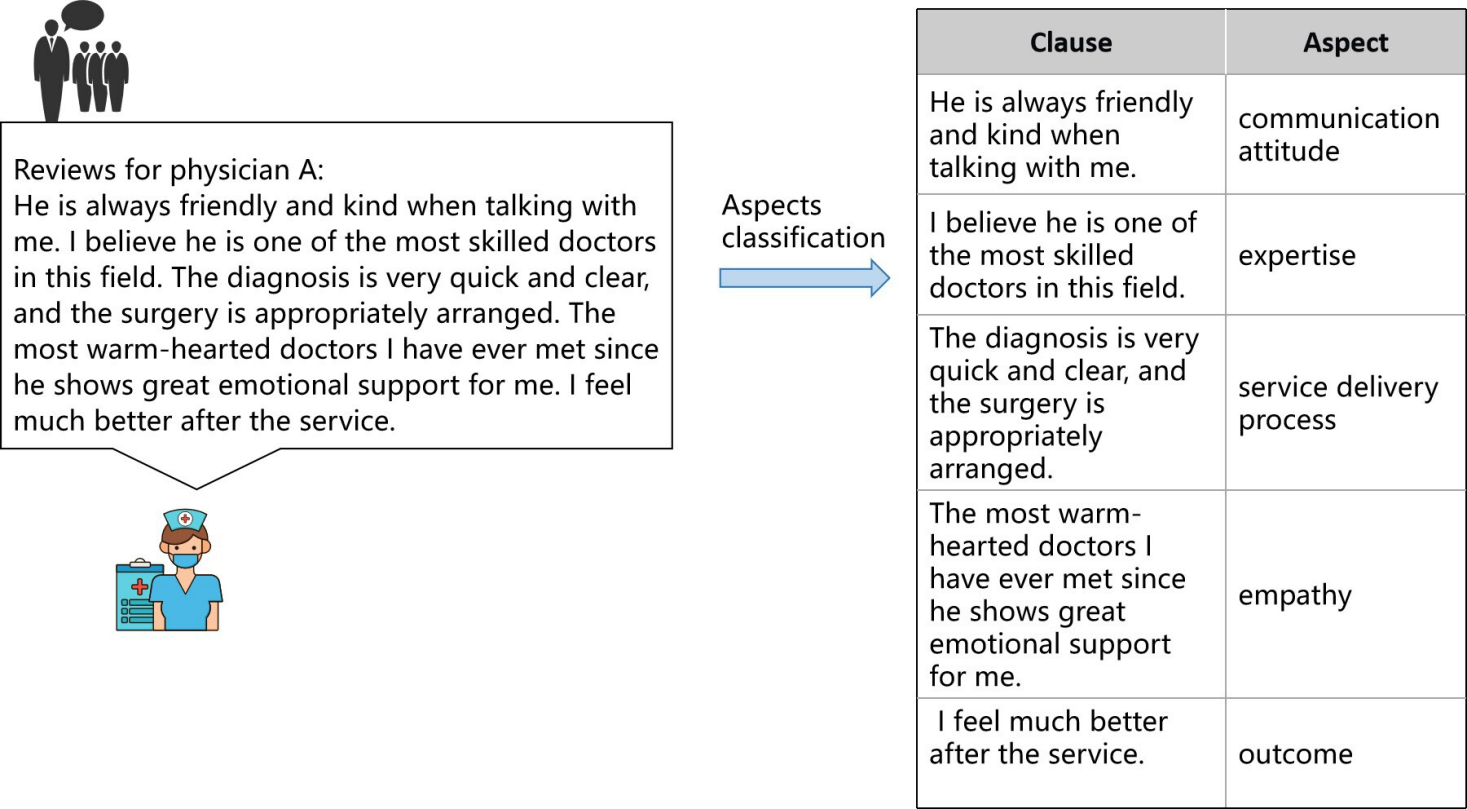
Example of aspects classification of reviews.

First, raw online reviews were segmented into clauses using an automatic procedure according to the semantic context and punctuation marks. After applying our program, 5,477,856 clauses were extracted from 672,195 reviews. We recruited domain experts and coded a labeled training set clause. Domain experts have extensive experience in using online healthcare platforms and text proofreading. A total of 103,726 clauses were randomly selected, and the experts were asked to code these clauses into 6 categories (including 5 quality dimensions and 1 category of “others”). Each clause was assigned to 2 experts to ensure its validity. We used Cohen kappa to assess interannotator reliability, yielding a final score of 0.93 (*P*<.001). As noted in previous literature [85], a Cohen kappa score above 0.8 indicates strong agreement between the annotators on the clause labeling task. Finally, we obtained a gold standard sample containing 84,733 clauses. Figure 3 shows an example of a coded clause from a review.

The clauses were then classified into different quality dimensions using a supervised classification approach called FastText [60]. To assess the performance of this classification model, we divided the labeled corpus into a training set and a test set for a double check (with 80% of the labeled set serving as the training set and the remaining 20% serving as the test set). The model parameters were optimized with a grid search to increase the prediction accuracy. Eventually, the model was set up with a learning rate of 0.8, a training count of 25, and 20 vector dimensions. In Table S3 in Multimedia Appendix 1, we compare the prediction performance of this model with a few common baselines, such as random forest and SVM. We also used a BERT (Bidirectional Encoder Representation from Transformers) model for the Aspect Classification task, setting the epoch to 3 and the learning rate to 2e-5. On a CPU, the task took three days to complete, whereas on a GPU, training took 32 minutes, with prediction requiring 0.73 minutes. The BERT model achieved an accuracy of 0.83. In comparison, our model achieved a performance of 0.93 within a reasonable amount of time and computational resources, which we consider sufficient and appropriate for our research.

The trained model was used to process all 5,477,856 clauses from 672,195 reviews according to the 5 proposed dimensions. Eventually, we obtained 871,522 (process), 795,568 (attitude), 563,312 (expertise), 299,663 (outcome), and 135,032 (empathy) clauses for each of the service quality dimensions. In total, 2,812,759 clauses could not be mapped to any category.

#### Sentiment Assessment

We then adopted a lexicon-based sentiment calculation approach to assess whether each clause contained the patients’ positive or negative experiences. Based on the sentiment lexicon HowNet [86], which contains negative, positive, and degree lexicons, the sentiment scores for each service quality dimension are calculated following the steps: (1) identify sentiment word in the clause, match it with the sentiment dictionary, and record its score and position; (2) find degree adverbs preceding the sentiment word, match them with the degree lexicon, and adjust the sentiment score by the weight of the adverbs; (3) check for negative words before the sentiment word, and if the number of negative words is odd, update the sentiment score accordingly; and (4) calculate the sentiment score by synthesizing the positive and negative sentiment values for each clause. After deriving the sentiment of each clause, we aggregated the sentiment value for each doctor in each quality dimension and then normalized it by averaging the score according to the number of words in the corpus.

### Ethical Considerations

This study qualifies for exemption from ethics review according to Article 32 (a) and (b) of the “Ethical Review Measures for Life Science and Medical Research Involving Humans” issued by the Ministry of Science and Technology of China [87], as it meets all required criteria: (1) our study does not involve direct interaction with human participants, poses no risk of physical harm and does not involve sensitive personal information or commercial interests and (2) the data we used are secondary data obtained from legitimate public online health platform (Haodf.com) and were properly anonymized. Under these provisions, formal institutional review board approval is not required.

## Results

In real-world applications, the above framework must be applied to the data of each doctor using text classification and sentiment calculation. In this study, we apply the research design and illustrate each procedure using our collected dataset.

### Dimension Extraction Results

**Figure 4.**
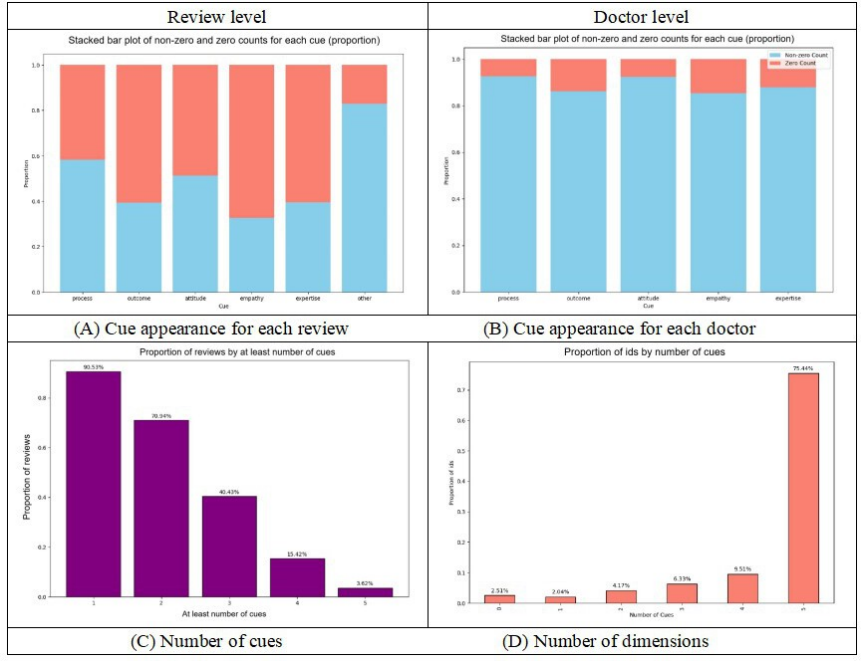
Cue distribution among reviews (A and C) and doctors (B and D).

Drawing on the extraction techniques, we can directly demonstrate the fine-grained service quality dimensions in the reviews. Figure 4 presents the distribution of the 5 cue dimensions among the reviews and doctors in our dataset.

At the review level, each review had an approximately 50% chance of obtaining opinions on each dimension. Specifically, 58% (389,873/672,195) of individual reviews contained the “service delivery process” cue; around 40% (268,878/672,195) of reviews included the “outcome” cue. In addition, approximately 84% (564,644/672,195) of the reviews mentioned content beyond the 5 specified cues. We examined the number of dimensions appearing in each review. Of individual reviews, 90.53% (608,538/672,195) mentioned at least 1 cue, 70.9% (476,586/672,195) contained more than 2 types of cues, and only 3.62% (24,333/672,195) of individual reviews included all 5. This suggests that most reviews tend to be concentrated on a small number of service aspects, and comprehensive reviews covering all cues are relatively rare.

However, the aggregated patterns at the doctor-level were slightly different. As we can see in Figure 4, the “service delivery process” cue was mentioned in approximately 85% (4186/4925) of doctors’ reviews, the “outcome” cue appeared in about 90% (4432/4925) of doctors’ reviews, and “attitude features” in 88% (4334/4925) of doctors’ reviews. In terms of the number of dimensions mentioned in each doctor’s review, approximately 97.48% (4801/4925) of doctors mentioned at least 1 cue, and 75.43% (3715/4925) of doctors mentioned all 5 cues in the reviews. Clearly, the 5 dimensions developed in our HSQ-5D framework account for a significant number of patients’ concerns regarding doctors.

### Validation Data Descriptive Statistics

We provide descriptive statistics for the variables in the econometric model in Table S4 in Multimedia Appendix 1. In terms of the 5 dimensions of doctor service quality, we derived—expertise, service delivery process, communication attitude, empathy, and outcome—patients perceive higher quality in doctors’ communication attitude and expertise, with scores of 0.06 and 0.04, respectively. This indicates that patients highly value the friendliness, professional competence, and knowledge levels of doctors in online services. However, the perception of treatment outcomes is lower, at 0.03. This suggests that patients were less satisfied with the treatment effects, possibly because they were not ideal or did not meet expectations.

While the average doctor serves 1377 patients, the actual number varies significantly across doctors. When evaluating doctors, patients also consider other factors such as doctors’ ranks, gender, ratings, and popularity as reflected by their homepage browsing, votes, thank-you letters, and virtual gifts. The doctor ranks include resident doctor, attending doctor, associate chief doctor, and chief doctor. In our dataset, 49.58% (2442/4925) of doctors were chronic disease doctors and 34.86% (1717/4925) were female doctors. The average rating for doctors was 3.72 (SD 0.58), reflecting the overall satisfaction of patients with doctors. The average number of views per doctor’s homepage was approximately 1 million. On average, each doctor received 89 votes, 34 thanks-you notes, and 83 virtual gifts. In the model, we also controlled the grade, type, and city of doctors’ hospitals.

The correlation matrix of the data is presented in Table S5 of the Multimedia Appendix 1. The primary variables in this study do not exhibit multicollinearity issues, indicating that the variables are sufficiently independent of one another, ensuring that the regression analyses and other statistical tests yield reliable and interpretable results.

### Regression Results

The regression results reinforce the framework’s relevance in digital health care contexts, where online reviews serve as a proxy for service quality assessments. By empirically linking key components in the HSQ-5D model to real-world doctor selection behavior, our study bridges the gap between theoretical service quality frameworks and practical patient decision-making. This methodological rigor strengthens the validity of the HSQ-5D framework as a tool for both researchers and practitioners seeking to understand and improve patient-centered care in online health care platforms.

Table 1 shows that all the dimensions derived from the patient reviews showed a significantly positive effect on their choices of doctors: expertise (coefficient=1.12; *P*<.001), communication attitude (coefficient=0.82; *P*<.001), service delivery process (coefficient=5.60; *P*<.001), and empathy (coefficient=2.65; *P*<.001). In the regression formula, the dependent variable, patient demand, was log-transformed. Therefore, for a given independent variable, an increase of 1 SD from its mean, the corresponding change in the number of patients can be calculated by multiplying the regression coefficient by the SD. For example, if the expertise dimension increases by 1 SD (0.03) from its mean, the log value of the patient count increases by 1.1189×0.0252≈0.03. This translates into an approximate increase of 2.9% (*e^0.0282^*‐1). The improvement of professional expertise not only improves patients’ health expectations but also enhances their trust and reliance on doctors. This underscores the significant impact of professional skill enhancement in attracting more patients. Similarly, if the service delivery process increases by one standard deviation (0.02) from its mean, the log value of the patient count increases by 5.5977×0.0210≈0.12. In other words, improvements in the service delivery process dimension, such as more efficient procedures and better workflow management, could increase patient loyalty and willingness to recommend, leading to an impressive 12.5% increase in the number of patients. Fostering a positive attitude by 1 SD (0.04) from the mean results in an increase of approximately 3.2% in the number of patients, emphasizing the significant role of a positive and supportive environment in enhancing patient satisfaction and retention. This indicates that a positive and compassionate manner can make patients feel valued and respected, encouraging them to trust their health care provider more and return for future visits. An increase in empathy by 1 SD led to an increase in the number of patients by approximately 7.3%, while a 1 SD increase in outcome led to a 2.5% increase, demonstrating the critical value of emotional intelligence and empathetic care in healthcare and highlighting the importance of achieving positive health results to attract and retain patients.

To further validate the robustness of our findings, 4 additional analyses were conducted, as detailed in Table S6 in Multimedia Appendix 1. First, Poisson regression and generalized least squares models were estimated. The coefficients of all service quality dimensions in both models are significantly positive, aligning with the main results. Second, to mitigate the influence of potential outliers, we censored the top and bottom 10% of observations for dependent variables. The robustness check yielded results consistent with the primary findings, confirming the stability of our model. Third, additional control variables were included in the analysis, including doctor’s department, number of visits to a hospital’s homepage, rank of the hospital, and number of patients served by the hospital. The results are consistent with the main results. Fourth, we replaced the original sentiment calculation steps with the BERT sentiment analysis model to capture the nuanced clause-level sentiment. The aggregated sentiment for each dimension is calculated based on each corresponding clause. The final values are also normalized based on the corpus length to obtain the alternative measurements for the 5 dimensions in the model. The test results align with the findings of our primary empirical analysis.

**Table 1.** Regression results from the econometric evaluation of HSQ-5D (5-dimensional health care service quality framework) dimensions.

Variables	Coefficient	SD	*P* value
Expertise	1.12	0.09	<.001
Process	5.60	0.23	<.001
Attitude	0.82	0.05	<.001
Empathy	2.65	0.14	<.001
Outcome	0.26	0.03	<.001
Chronic	−0.14	0.03	<.001
Rank	−0.08	0.01	<.001
Gender	0.045	0.02	.018
log (rating)	−0.15	0.01	<.001
log (visit)	0.53	0.01	<.001
log (vote)	0.14	0.004	<.001
log (thanks)	0.21	0.01	<.001
log (gifts)	0.25	0.01	<.001
Constant	−2.52	0.16	<.001

### Information Load Results

To further validate the value of the 5 quality dimensions, we used a stepwise feature addition approach using a panel data linear regression incorporating each of the 5 dimensions (expertise, service delivery process, communication attitude, empathy, and outcome) one by one. As shown in Table S7 in Multimedia Appendix 1, this leads to an increase in *R*^2^ from 79% to 85%, with each feature contributing to an increase of 1% to 3%. Given the limited ability of linear regression, it is clear our developed dimensions capture essential information related to patients’ selection.

In addition, we evaluated the model by including all text from reviews beyond the 5 specified dimensions. Specifically, we vectorized the additional text and incorporated it directly into the linear regression model without performing complex calculations. The results indicated that the performance only changed by 0.1% from that of our model with 5 dimensions, which is clearly not a major improvement. This suggests that the 5 dimensions of our framework have captured the most essential information in online reviews for predicting patient selection for doctors.

## Discussion

### Principal Findings

We developed HSQ-5D, a text-mining-based health care service quality assessment framework that leverages patient online reviews to extract insights into doctors’ service quality dimensions. The framework was designed for real-world application through text classification and sentiment analysis. Using data from a large online health care platform in China, we implement the framework and conduct an empirical study to validate whether the extracted dimensions can predict demand (since they reflect doctors’ service quality). Based on the 672,195 reviews of 4925 doctors, we found that 75.43% (3715/4925) of doctors had reviews covering all 5 dimensions, indicating that the HSQ-5D framework effectively captures key patient concerns about doctors. Regression analysis further confirmed that the 5 identified dimensions—expertise, service delivery process, communication attitude, empathy, and outcome—significantly explain variations in the demand for health care services. Specifically, an increase of 1 SD in expertise leads to a 2.9% rise in patient demand. A 1 SD increase in the service delivery process results in a 12.5% boost in demand. A positive attitude increases patient demand by 3.2%, while empathy contributes to a 7.3% increase. Improved outcomes yield a 2.5% rise in patient demand. Furthermore, a stepwise feature addition approach showed that including all 5 dimensions raised the model’s *R*^2^ from 79% to 85%, emphasizing the importance of these dimensions in capturing essential factors that influence patient decision-making. This further underscores the HSQ-5D frameworks’ ability to extract essential quality information from reviews comprehensively.

### Comparison With Previous Work

It is worthwhile to compare our framework with the existing quality assessment frameworks. In particular, Donabedian’s SPO model and SERVQUAL are two widely used quality assessment frameworks. SPO is primarily designed for traditional health care services and has limited applicability in emerging fields such as online health care or digital health. However, it is qualitative and lacks specific quantitative metrics for measuring quality. SERVQUAL often relies on questionnaires to assess service quality, which can be restricted to small samples and typically require manual completion and data organization. The HSQ-5D framework enables the quality assessment of online platforms through rapid and efficient information extraction and processing. This significantly lowers the barriers to data collection and the analysis of health care services.

We also conduct empirical comparisons of the HSQ-5D framework against SPO and SERVQUAL through a systematic variable alignment. In the SPO model, we maintain identical constructs for process and outcome dimensions while mapping structure to control variables such as hospital type, location, grade, and doctor designation and rank. According to Table S7 in Multimedia Appendix 1, this configuration resulted in an *R*^2^ of 0.83. For the SERVQUAL model, we preserved the empathy dimension while mapping responsiveness to the service delivery process dimension and reliability to expertise, with assurance and tangibles treated using several control variables. This alignment yielded *R*^2^ of 0.83. Our HSQ-5D framework demonstrated superior performance with an *R*^2^ of 0.86, outperforming both existing models. The incremental contribution of 1% to 3% in explanatory power from each dimension indicates their high accuracy and sensitivity in capturing the factors most relevant to patient selection regarding service quality. Furthermore, incorporating additional unstructured data from reviews produced only a negligible 0.1% improvement in the model performance. This empirical investigation further reinforces that our framework comprehensively captures the most critical aspects of health care service quality as reflected in patient reviews.

### Strengths and Limitations

#### Theoretical Implications

This study makes several significant theoretical contributions. First, we proposed the HSQ-5D Model, which systematically integrates 5 quality dimensions (the expertise of doctors, service delivery process, communication attitude, empathy, and the outcomes achieved) from online reviews to provide insights into the quality of health care services that influence patient decisions. Our empirical analysis demonstrates that these quality dimensions have a significant impact on patient demand. By structuring unstructured patient feedback, the HSQ-5D Model provides a comprehensive lens through which to analyze patients’ perceptions of health care services. This model advances existing literature and offers a more nuanced understanding of how patients evaluate health care providers in the digital age.

Second, we introduce an innovative text-mining approach to quantitatively analyze unstructured online review data. This methodological contribution enables the extraction and construction of service quality–related variables, transforming extensive and complex social media data into actionable insights. While developed in a health care context, our methodology offers broader applicability for analyzing online reviews across various service industries. This advancement provides researchers with a novel procedure for deriving theoretical constructs from large-scale textual data, encouraging new directions for digital content analysis in management research.

#### Practical Implications

This study provides practical ideas for health care stakeholders by emphasizing the critical need to enhance health care services for the benefit of all parties involved. This study contributes to the establishment of a more effective healthcare ecosystem by deepening the understanding of patient decision-making behaviors and identifying challenges within health care services. These findings have the potential to improve public health services, relieve the pressure on existing health care systems, and propose a user-centric and adaptive health care ecosystem in underdeveloped countries that effectively integrates limited medical resources for value creation.

Our study also provides online health care platforms and doctors with actionable ideas for implementing excellent health care services. For medical platforms, a central recommendation involves refining the presentation of health care information to aid patients in comprehending 5 core quality dimensions. Online platforms can extract and highlight these dimensions to assist patients. Doctors can enhance patient satisfaction and trust by systematically monitoring and evaluating the 5 quality dimensions. The effective management of patient feedback ensures that services are consistently aligned with patient desires, thereby improving overall service quality and attracting potential patients.

#### Limitations

Several limitations should be acknowledged and addressed in future research. First, patient expectations and perceptions of health care quality can vary significantly across diverse cultural contexts and health care systems. Factors such as cultural norms, regional customs, and variations in the health care infrastructure influence how patients interpret and prioritize service quality dimensions. Customized quality dimensions tailored to the focal culture can be introduced in future research to address this cross-cultural issue. In addition, the availability of healthcare resources and services in different countries may impact how patients assess the quality of medical services. Based on the specific characteristics of each healthcare system, the weights assigned to certain dimensions within the framework can be adjusted. Second, although FastText offers efficient and scalable text representations, it lack the ability to fully leverage the contextual dependencies present in longer texts. To address this limitation, we suggest exploring alternative machine learning methods in future research, such as transformer-based or large-language models (eg, BERT or GPT), which provide more sophisticated contextual embeddings and attention mechanisms.

### Conclusion

This study advances the theoretical discourse on digital health care by introducing a comprehensive framework for assessing service quality, using innovative analytical methods, and elucidating the influence of contextual factors on patient decision-making. The findings also offer practical guidance for health care providers and platforms to enhance service quality and better meet patient needs.

## Supplementary material

10.2196/66141 Multimedia Appendix 1 Additional tables and figures.

## Abbreviations

AI: artificial intelligence
BERT: Bidirectional Encoder Representation from Transformers
HSQ-5D: 5-dimensional health care service quality framework
LDA: latent Dirichlet allocation
SERVQUAL: service quality
SPO: structure-process-outcome
