# Supplementary material for: Developing a Framework for Online Review-Based Health Care Service Quality Assessment: Text-Mining Study
Source: J Med Internet Res. 2025 Jul 9;27:e66141. doi: 10.2196/66141 (PMC12266612; doi:10.2196/66141)
Supplement: Multimedia Appendix 1 [file jmir-v27-e66141-s001.docx]

# Multimedia Appendix 1

## Additional Figures and Tables

**Table S1. Topic Extraction Result and the Sample Key Words in Each Topic**

| Topic 1:  Operational Expertise | Topic 2: Service Attitude | Topic 3: Technical Process | Topic 4: Patient Empathy | Topic 5: Care Approach | Topic 6: Careful Process | Topic 7: Recovery Outcome | Topic 8: Empathetic Service | Topic 9: Professionalism | Topic 10: Pain Management Outcome | Topic 11: Chronic Care Outcome | Topic 12: Treatment Effectiveness |
| --- | --- | --- | --- | --- | --- | --- | --- | --- | --- | --- | --- |
| operation | attitude | reply | patience | professional | careful | recover | goodness | professor | pain | chronic | repair |
| expertise | graveness | technique | solution | relative | kind | seek | kind | brilliant | relieve | normal | hope |
| successful | responsible | elaborate | advice | plan | warm | clear | beautiful | scheme | exercise | stable | bright |
| superb | patients | group | consultation | care | patience | improve | angle | benevolence | symptom | control | thank |
| smooth | ethics | arrangement | treatment | examine | disease | medicine | warmth | help | lighten | Medication | great |
| skill | service | total | check | treat | sincerity | prescribe | sister | experience | effect | experiment | risk |
| recover | enthusiastic | Infusion | diagnosis | response | humorous | symptomatic | voice | appreciation | livelihood | indicator | rebirth |
| arrangement | kindness | discover | therapy | approach | intimate | dose | spring | give | senior | drop | satisfied |
| specialist | experience | deserve | condition | skill | meticulous | medical | heart | life | recidivation | dose | natural |
| procedure | gracious | opportunity | question | consummate | goodness | doctor | goodness | analysis | terrible | cure | expectation |

**Table S2. The Topic Mapping Results**

| **Topic Number** | **Expert 1’s Rating** | **Expert 2’s Rating** |
| --- | --- | --- |
| Topic 1: Operational Expertise | Doctors’ Expertise | Doctors’ Expertise |
| Topic 2: Service Attitude | Communication Attitude | Communication Attitude |
| Topic 3: Technical Process | Service Delivery Process | Service Delivery Process |
| Topic 4: Patient Empathy | Empathy | Empathy |
| Topic 5: Care Approach | Service Delivery Process | Service Delivery Process |
| Topic 6: Careful Process | Service Delivery Process | Service Delivery Process |
| Topic 7: Recovery Outcome | Outcome of Services | Outcome of Services |
| Topic 8: Empathetic Service | Empathy | Empathy |
| Topic 9: Professionalism | Doctors’ Expertise | Doctors’ Expertise |
| Topic 10: Pain Management Outcome | Outcome of Services | Outcome of Services |
| Topic 11: Chronic Care Outcome | Outcome of Services | Outcome of Services |
| Topic 12: Treatment Effectiveness | Outcome of Services | Outcome of Services |

**Table S3. Comparative Performance Evaluation of Multiple Algorithms in Aspect Classification**

| Algorithm | Training time (min) | Forecasting time (min) | Accuracy |
| --- | --- | --- | --- |
| FastText | 6.0 | 0.06 | 0.93035 |
| BERT | 32.11 | 0.73 | 0.8270 |
| Bagging | 31.39 | 0.98 | 0.5648 |
| TREE | 1.22 | 0.665 | 0.6622 |
| GLM | 2.42 | 0.6 | 0.4507 |
| NNET | 102.34 | 0.72 | 0.6642 |
| RandomForest | 360.61 | 1.12 | 0.5783 |
| SVM | 59.69 | 2.56 | 0.6021 |

**Table S4. Summary Statistics**

| Variables | Observations | Mean | SD | Minimum | Maximum | Definition |
| --- | --- | --- | --- | --- | --- | --- |
| Expertise | 58,627 | 0.0444 | 0.0252 | -0.1661 | 0.2584 | The sentiment value of the expertise of a doctor |
| Process | 58,973 | 0.0311 | 0.0210 | -0.2069 | 0.2364 | The sentiment value of the service delivery process |
| Attitude | 58,893 | 0.0646 | 0.0384 | -0.3404 | 0.6709 | The sentiment value of the communication attitude of a doctor |
| Empathy | 58,398 | 0.0386 | 0.0266 | -0.1688 | 0.2506 | The sentiment value of the empathy of a doctor |
| Outcome | 58,475 | 0.0306 | 0.0934 | -0.7944 | 0.3416 | The sentiment value of the service outcome |
| Patients | 59,328 | 1,377 | 2,940 | 2 | 46,424 | The number of patients served by a doctor |
| Chronic | 59,328 | 0.4959 | 0.4999 | 0 | 1 | Equals 1 if the doctor is from a department of chronic disease and 0 otherwise |
| Gender | 59,328 | 0.3487 | 0.4765 | 0 | 1 | Equals 1 if the doctor is female and 0 otherwise |
| Rating | 59,328 | 3.7195 | 0.5821 | 1 | 5 | A doctor’s rating is between 1 and 5 |
| Visit | 59,328 | 1,041,164 | 4,679,697 | 77 | 1,926,356,423 | The number of views for a doctor’s homepage |
| Vote | 59,328 | 89 | 162 | 0 | 2,970 | The number of votes received by a doctor |
| Thanks Letter | 59,328 | 34 | 77 | 0 | 1,598 | The number of thanks letters received by a doctor |
| Gift | 59,328 | 83 | 248 | 0 | 6,314 | The number of virtual gifts received by a doctor |
| Rank | 59,184 | 2.955 | 0.928 | 1 | 4 | The Rank of a doctor in the hospital |

**Table S5. Correlations Matrix**

| Variables | (1) | (2) | (3) | (4) | (5) | (6) | (7) | (8) | (9) | (10) | (11) | (12) | (13) | (14) |  |
| --- | --- | --- | --- | --- | --- | --- | --- | --- | --- | --- | --- | --- | --- | --- | --- |
| (1) Patients | 1.000 |  |  |  |  |  |  |  |  |  |  |  |  |  |  |
| (2) Expertise | 0.057*** | 1.000 |  |  |  |  |  |  |  |  |  |  |  |  |  |
| (3) Process | -0.034*** | 0.064*** | 1.000 |  |  |  |  |  |  |  |  |  |  |  |  |
| (4) Attitude | 0.021*** | 0.140*** | 0.119*** | 1.000 |  |  |  |  |  |  |  |  |  |  |  |
| (5) Empathy | 0.071*** | 0.280*** | 0.075*** | 0.120*** | 1.000 |  |  |  |  |  |  |  |  |  |  |
| (6) Outcome | -0.005 | 0.034*** | 0.028*** | 0.024*** | 0.038*** | 1.000 |  |  |  |  |  |  |  |  |  |
| (7) Chronic | -0.178*** | -0.063*** | 0.066*** | -0.037*** | -0.046*** | -0.016*** | 1.000 |  |  |  |  |  |  |  |  |
| (8) Gender | -0.119*** | -0.052*** | 0.046*** | -0.026*** | -0.018*** | 0.005 | 0.738*** | 1.000 |  |  |  |  |  |  |  |
| (9) Rating | 0.207*** | 0.057*** | -0.027*** | -0.024*** | 0.073*** | 0.006 | -0.057*** | -0.046*** | 1.000 |  |  |  |  |  |  |
| (10) Visit | 0.708*** | 0.017*** | -0.035*** | -0.011*** | 0.033*** | -0.009** | -0.089*** | -0.056*** | 0.085*** | 1.000 |  |  |  |  |  |
| (11) Vote | 0.713*** | 0.074*** | -0.042*** | 0.032*** | 0.091*** | 0.008* | -0.162*** | -0.112*** | 0.337*** | 0.378*** | 1.000 |  |  |  |  |
| (12) Thanks Letter | 0.655*** | 0.054*** | -0.044*** | 0.016*** | 0.067*** | 0.008* | -0.132*** | -0.092*** | 0.336*** | 0.345*** | 0.972*** | 1.000 |  |  |  |
| (13) Gift | 0.747*** | 0.034*** | -0.035*** | 0.011*** | 0.051*** | 0.000 | -0.136*** | -0.094*** | 0.233*** | 0.466*** | 0.826*** | 0.801*** | 1.000 |  |  |
| (14) Rank | 0.092*** | 0.054*** | -0.056*** | -0.006 | 0.051*** | 0.008** | -0.009** | 0.002 | 0.179*** | 0.057*** | 0.121*** | 0.103*** | 0.085*** | 1.000 |  |

**Table S6. Robust Check Results**

|  | Variables | Coef. | SD | *P* value |
| --- | --- | --- | --- | --- |
| Poisson | **Expertise** | .6792 | 0.1036 | <.001 |
|  | **Process** | 1.0447 | 0.1241 | <.001 |
|  | **Attitude** | .5788 | 0.0651 | <.001 |
|  | **Empathy** | 2.6468 | 0.1003 | <.001 |
|  | **Outcome** | .0431 | 0.0236 | .068 |
| GLS | **Expertise** | 2.1197 | 0.1492 | <.001 |
|  | **Process** | 4.2364 | 0.1712 | <.001 |
|  | **Attitude** | 2.7718 | 0.0976 | <.001 |
|  | **Empathy** | 2.1478 | 0.1430 | <.001 |
|  | **Outcome** | .1574 | 0.0367 | <.001 |
| OLS (Censored data) | **Expertise** | 1.0824 | 0.0913 | <.001 |
|  | **Process** | 5.2749 | 0.2361 | <.001 |
|  | **Attitude** | .9403 | 0.0586 | <.001 |
|  | **Empathy** | 2.6429 | 0.1448 | <.001 |
|  | **Outcome** | .2256 | 0.0475 | <.001 |
| OLS (More controls) | **Expertise** | .8950 | 0.0902 | <.001 |
|  | **Process** | 4.2358 | 0.2290 | <.001 |
|  | **Attitude** | .5536 | 0.0522 | <.001 |
|  | **Empathy** | 1.8905 | 0.1380 | <.001 |
|  | **Outcome** | .1888 | 0.1615 | .242 |
| OLS（Bert Sentiment） | **Expertise** | 1.307 | 0.380 | <.001 |
|  | **Process** | 1.980 | 0.289 | <.001 |
|  | **Attitude** | 3.885 | 0.259 | <.001 |
|  | **Empathy** | 1.122 | 0.477 | 0.0187 |
|  | **Outcome** | 0.121 | 0.0495 | 0.0143 |

Table S7. Performance Comparison of Multiple Prediction Models (*P*<0.01 ***, *P*<0.05 **, *P*<0.1 *）

| **Gradually Add Features Models** | | | | | | | **SPO** | **SERVQUAL** | **All Text** |
| --- | --- | --- | --- | --- | --- | --- | --- | --- | --- |
| **Process** |  |  |  |  |  | 5.5977^***^ | 6.891^***^ | 6.198^***^ | 4.4294^***^ |
|  |  |  |  |  |  | (0.227) | (0.195) | (0.195) | (0.2475) |
| **Expertise** |  |  |  |  | 1.4116^***^ | 1.1189^***^ |  | 1.566^***^ | 1.0368^***^ |
|  |  |  |  |  | (0.104) | (0.0906) |  | (0.0813) | (0.1) |
| **Empathy** |  |  |  | 3.1908^***^ | 3.0127^***^ | 2.6468^***^ |  | 2.924^***^ | 2.0840^***^ |
|  |  |  |  | (0.147) | (0.1464) | (0.137) |  | (0.117) | (0.1458) |
| **Attitude** |  |  | 1.0015^***^ | 0.9025^***^ | 0.8613^***^ | 0.8232^***^ |  |  | 0.5564^***^ |
|  |  |  | (0.0599) | (0.0589) | (0.0585) | (0.0532) |  |  | (0.0561) |
| **Outcome** |  | 0.3829^***^ | 0.3535^***^ | 0.3072^***^ | 0.2913^***^ | 0.2626^***^ | 0.464^***^ |  | 0.1692^***^ |
|  |  | (0.0557) | (0.0347) | (0.0385) | (0.0468) | (0.0264) | (0.0540) |  | (0.0447) |
| **Chronic** | -0.3042^***^ | -0.3021^***^ | -0.3053^***^ | -0.3095^***^ | -0.3107^***^ | -0.1442^***^ | -0.0866^***^ | 2.924^***^ | -0.2600^***^ |
|  | (0.0308) | (0.0308) | (0.0308) | (0.0307) | (0.0307) | (0.0274) | (0.0264) | (0.117) | (0.0306) |
| **Rank** | -0.1047^***^ | -0.1031^***^ | -0.0976^***^ | -0.0943^***^ | -0.0931^***^ | -0.0822^***^ | -0.105^***^ | -0.0767^***^ | -0.0661^***^ |
|  | (0.0130) | (0.0130) | (0.0128) | (0.0127) | (0.0127) | (0.0125) | (0.0130) | (0.0257) | (0.0094) |
| **Gender** | 0.0450^**^ | 0.0428^*^ | 0.0401^*^ | 0.0348 | 0.0336 | 0.0451^**^ | 0.0574^***^ | -0.0990^***^ | 0.0281 |
|  | (0.0226) | (0.0228) | (0.0228) | (0.0227) | (0.0227) | (0.0191) | (0.0143) | (0.0129) | (0.0231) |
| **log(Rating)** | -0.2481^***^ | -0.2472^***^ | -0.2306^***^ | -0.2131^***^ | -0.2087^***^ | -0.1544^***^ | -0.123^***^ | 0.0192 | -0.1798^***^ |
|  | (0.0101) | (0.0101) | (0.0101) | (0.0101) | (0.0101) | (0.0094) | (0.00862) | (0.0128) | (0.0101) |
| **log(Visit)** | 0.5873^***^ | 0.587^***^ | 0.5831^***^ | 0.5767^***^ | 0.5750^***^ | 0.5302^***^ | 0.519^***^ | -0.112^***^ | 0.5201^***^ |
|  | (0.0061) | (0.0061) | (0.0060) | (0.006) | (0.006) | (0.0097) | (0.00725) | (0.00858) | (0.0065) |
| **log(Vote)** | 0.2069^***^ | 0.2061^***^ | 0.1985^***^ | 0.1868^***^ | 0.1819^***^ | 0.1420^***^ | 0.184^***^ | 0.516^***^ | 0.1526^***^ |
|  | (0.0044) | (0.0045) | (0.0045) | (0.0045) | (0.0044) | (0.0039) | (0.00270) | (0.00718) | (0.0044) |
| **log(Thanks)** | 0.0696^***^ | 0.0694^***^ | 0.0695^***^ | 0.0683^***^ | 0.0672^***^ | 0.2140^***^ | 0.256^***^ | 0.167^***^ | 0.0688^***^ |
|  | (0.0048) | (0.0048) | (0.0048) | (0.0048) | (0.0048) | (0.0087) | (0.00589) | (0.00274) | (0.0047) |
| **log(Gifts)** | 0.0778^***^ | 0.0777^***^ | 0.0778^***^ | 0.0770^***^ | 0.0765^***^ | 0.2525^***^ | 0.299^***^ | 0.244^***^ | 0.0793^***^ |
|  | (0.0038) | (0.0038) | (0.0038) | (0.0038) | (0.0038) | (0.0089) | (0.00638) | (0.00588) | (0.0038) |
| **R2** | 0.7928 | 0.8229 | 0.8360 | 0.8473 | 0.8489 | 0.8552 | 0.828 | 0.831 | 0.8567 |


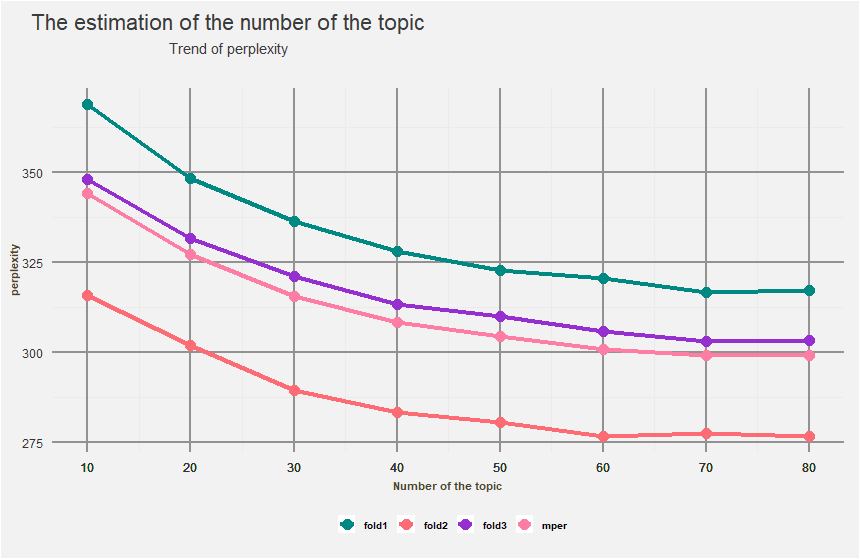

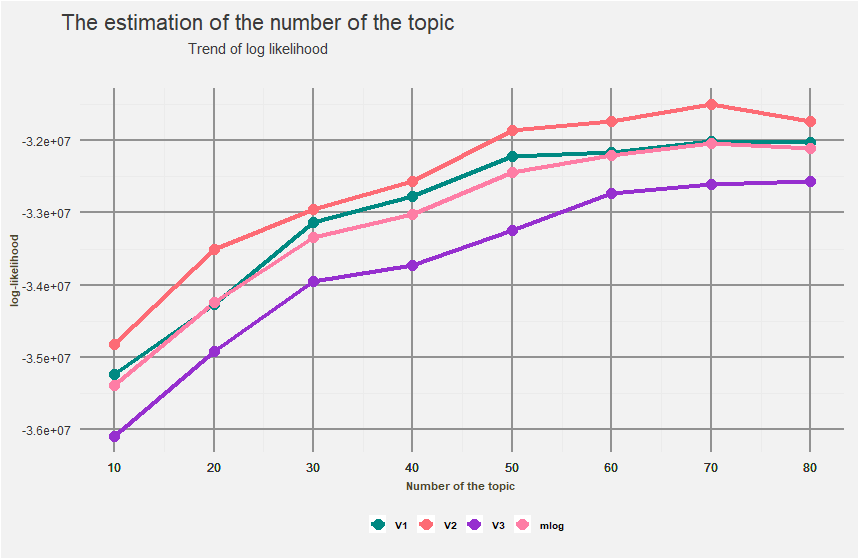


**Figure S1. The Performance Validation of LDA Topic Extraction**
